# Mobile Apps as Audience-Centered Health Communication Platforms

**DOI:** 10.2196/25425

**Published:** 2021-08-17

**Authors:** Michael Mackert, Dorothy Mandell, Erin Donovan, Lorraine Walker, Mike Henson-García, Lindsay Bouchacourt

**Affiliations:** 1 Stan Richards School of Advertising and Public Relations The University of Texas at Austin Austin, TX United States; 2 Center for Health Communication The University of Texas at Austin Austin, TX United States; 3 School of Community and Rural Health University of Texas Health Science Center at Tyler Tyler, TX United States; 4 Population Health The University of Texas System Austin, TX United States; 5 Department of Communication Studies The University of Texas at Austin Austin, TX United States; 6 School of Nursing The University of Texas at Austin Austin, TX United States; 7 School of Public Health University of Texas Health Science Center - Dallas Regional Campus Dallas, TX United States

**Keywords:** health communication, mHealth, mobile apps, mobile health, prenatal health, pregnancy, audience-centered

## Abstract

Health communication campaigns often suffer from the shortcomings of a limited budget and limited reach, resulting in a limited impact. This paper suggests a shift of these campaigns to audience-centered communication platforms—particularly, apps on mobile phones. By using a common platform, multiple interventions and campaigns can combine resources and increase user engagement, resulting in a larger impact on health behavior. Given the widespread use of mobile phones, mobile apps can be an effective and efficient tool to provide health interventions. One such platform is Father’s Playbook, a mobile app designed to encourage men to be more involved during their partner’s pregnancy. Health campaigns and interventions looking to reach expectant fathers can use Father’s Playbook as a vehicle for their messages.

## Introduction

There are numerous public health issues, ranging from disparities in maternal mortality to the reframing of child abuse as a population health concern, where communication plays a key part in the solution. For example, the US Centers for Disease Control and Prevention’s *Tips From Former Smokers* was a national mass media anti-smoking campaign that profiled real people living with serious long-term health effects from smoking [1]. The campaign is known for its television spots with graphic and emotional testimonials from former smokers. This massive campaign helped approximately 1 million people successfully quit smoking [1]. A health communication example using new media is the Text4Baby smartphone app, which is aimed toward expectant and new mothers. The app provides information on topics ranging from baby milestones to nutrition to childcare tips, and it sends over 250 SMS text messages to the user’s phone with the most critical information for pregnant women and mothers [2]. Women who used the Text4Baby app felt more prepared to be new mothers [3], had higher attitudes toward prenatal vitamins [4], and had a higher level of pregnancy health knowledge [4].

Although high-profile and effective health communication interventions exist, it must be acknowledged that many do not achieve their objectives. A primary reason could be that health communication interventions are “underdosed”—most simply do not have the budget to reach enough people to make a substantial difference. Although the *Tips From Former Smokers* campaign was very successful, it had a budget of US $48 million in 2012 [1], which is anomalously high among health campaign budgets. This applies to health communication campaigns that focus on broad message dissemination as well as to more complex and interactive interventions.

Health communication could benefit from a fundamental paradigm shift. Interventions are often designed appropriately by following best practices [5,6], working within the budget and evaluation constraints of a particular project. To improve the efficacy of health communication interventions, we suggest a shift to audience-centered communication platforms, which are platforms that focus on a specific audience but tailor their content to address different subject matter (in this case, health issues) or different subsets of the audience.

When considering the potential of an audience-centered platform, mobile apps specifically can be extremely effective due to their versatility in content and widespread reach. Mobile apps could provide opportunities for multiple interventions and campaigns to combine resources and increase user engagement in service of promoting health behavior change and public health. As just one example, Bright by Text, a smartphone app for parents that provides information about early childhood topics, has regional implementations [7]. These regional implementations provide location-specific activities for parents to do with their children; this geographic tailoring can make the content more specific and useful for parents who live in different regions across the United States and avoids the need for regional programs to find their own ways to spread information to those parents.

Given the widespread use of mobile devices in the general public [8], delivering health messages through mobile apps is particularly useful [9]. Mobile apps can efficiently deliver appropriate doses of health messages along with providing “in-the-moment” health interventions [10,11], which are essentially a type of just-in-time adaptive intervention [12]. Mobile apps can deliver tailored health messages to people in specific and opportune moments within their everyday settings, effectively addressing the “message dosing issues” many health campaigns face [10,11]. Mobile apps can also effectively intervene among multiple health issues in one specific group or population. Health communication interventions are often built around a single health issue, despite the fact that many health issues coincide together within specific groups of individuals [13-15]. The time and resources used on promoting many independent health communication interventions could be better spent if there was a common platform that could be shared, especially if the interventions were all targeting a common audience.

One such platform to share health information is Father’s Playbook, a smartphone app designed for men to use during and after their partner’s pregnancy. Health communication interventions and campaigns attempting to reach expectant fathers can use Father’s Playbook as a way to reach an audience that already exists.

## Father’s Playbook Case Study

The Father’s Playbook smartphone app was created to fill a large gap in pregnancy-related health information—the lack of male-focused pregnancy information and resources. While women should be the main focus during pregnancy, including men has benefits for father, mother, and baby. Father involvement during the prenatal stage has shown to have many positive outcomes, including increased communication between partners, higher number of provider visits during pregnancy, and increased postpartum best practices [16,17]. Although men do feel strongly about being involved in pregnancy health [18], structural health care barriers and personal barriers may prevent them from being engaged. To help combat these barriers, Father’s Playbook was designed to engage men in prenatal health.

The Father’s Playbook app was developed using an incremental approach to the research and the app’s development. This process was cost-effective, and it allowed us to learn from mistakes and build on previous knowledge. Before the Father’s Playbook smartphone app existed, a web-based pilot was created. To obtain more generalizable results regarding attitudes and barriers to prenatal health involvement, a survey with a nationally representative sample of men was conducted [18]. Overall, men believe that it is important to be involved in pregnancy health; however, perceived barriers (eg, time restraints, unclear role, financial burdens) still exist [18]. This survey also required participants to interact with the website, and the participants made suggestions on how to improve the site. Through interviews and the survey, we were able to obtain men’s opinions on the website, along with suggestions for features that would improve the website [19]. This information allowed us to prioritize features to be developed (articles and an interactive budget calculator) and focus on user testing for new features.

Both the app’s development and future research related to the app have next steps. Presently, the app’s content is available in English and Spanish, which opens the door to a larger audience of men. Following this type of audience specification, in the future, the goal is to tailor content toward different types of fathers. For example, the experience of a stay-at-home father versus a single parent father greatly differs, and the pregnancy and fatherhood experience of transgender men who become pregnant will differ from the experience of a cisgender man [20]. On the research side, the next step will be to focus on the father’s engagement in prenatal health in a broader sense. To be able to measure the effectiveness of Father’s Playbook in improving father engagement, baseline data is needed on the current level of father engagement in prenatal health. As such, a representative survey targeting fathers and expectant fathers is being conducted to gather baseline data. Future development of the app can strengthen its potential as a platform to enable more efficient communication with expectant fathers rather than individual programs and efforts to reach this audience.

There are a wide variety of opportunities to improve paternal, maternal, and child health through improved communication with expectant fathers. There is a wealth of evidence showing that fathers want to be more engaged in the pregnancy and feel underprepared for fatherhood [19,21,22]. However, there is a dearth of research focused on how to engage and prepare fathers. The app provides a flexible platform to test education and communication strategies with fathers directly. For instance, directed education about nutrition and pregnancy complications may result in men assisting their pregnant partners in making healthier nutrition choices and may increase their ability to identify obstetric emergencies. Evidence also suggests that when expectant parents are unsatisfied with their partnerships, they are more likely to exhibit insensitive parenting styles once their infant is born. The app may provide a platform for disseminating communication interventions to help the quality of the relationship between expecting couples [23]. Additionally, studies have found that low levels of paternal engagement throughout the prenatal period are related to birth complications [24] and to low postnatal engagement. Positive father involvement in early childhood is tied to a variety of positive cognitive and health outcomes for children [25]; therefore, increasing the father’s engagement has the potential to result in positive health outcomes across the entire family unit.

## Father’s Playbook as an Audience-Centered Platform

Father’s Playbook was designed using audience-centered principles, which means that the content, language, and approach used in the app are tailored to best fit the audience (new and expectant fathers). By focusing on the audience, the app can tailor information and its delivery to the needs of the audience. In its current format, Father's Playbook is a single app that includes content pages about specific father-related information and interactive app features (such as a budget calculator) to encourage expectant fathers to become more engaged during and after their partner’s pregnancy. Future versions of the app will include tailored content, allowing the app to be more personally relevant to its user. This ability to tailor content can allow other health communication scholars to use the app to deploy tailored interventions and campaigns to the target audience of expectant and new fathers.

The shift to thinking of audience-centered platforms can broaden the reach and efficiency of interventions that have successfully developed an approach for reaching a particular audience. As an example, although Father’s Playbook is currently focused on amplifying the engagement of expectant fathers during the prenatal period, there are other issues that commonly affect expectant fathers, and at any time, the app can deploy other types of content to address the audience’s other public health needs. Other examples of public health information expectant fathers might want to consume include management of nutrition and physical activity throughout pregnancy, prevention of paternal postpartum depression, smoking cessation, and information related to paternity testing. These different health issues can be addressed specifically through tailored content in the app, without the need for researchers and professionals with interests in these issues to develop an entirely new intervention or campaign and then determine how to reach the target audience.

Along with addressing health issues of expectant and new fathers, Father’s Playbook can be used to address parenting issues and child-rearing best practices. For example, a researcher could be interested in testing an intervention that increases the amount of time a father reads to his children; as part of the intervention, participants would need to download the Father’s Playbook app. The app could add features that specifically work to reach the intervention objectives, such as content articles that discuss the benefits of fathers reading to their children, interactive games that encourage book reading, and a list of book suggestions appropriate for specific age ranges.

Father’s Playbook was built with the goal of increasing father engagement during pregnancy. Now that an audience of expectant and new fathers exists, other researchers and practitioners can access this unique audience to address a myriad of parenting and health issues, allowing a collaborative approach toward health communication campaigns and interventions. Without the use of an audience-centered platform, health professionals would only need to use their own resources to reach this audience.

## Conclusion

Health communication can be an effective tool to help improve the health and well-being of individuals and populations. There is a strong evidence base of health communication that can be leveraged across health issues and audiences, such as the increased efficacy of targeted and tailored messages compared to more general appeals [26].

Despite the high-profile success of health communication campaigns that have achieved important and demonstrated benefits, substantial opportunities remain to advance the field through new approaches to message design and reaching audiences. The approach advocated in this paper is to focus on more audience-focused platforms, which could be a more efficient strategy for message dissemination. Taking an audience-centered approach allows for a better understanding of people, their behaviors, their contexts, and their intersections allowing for more nuanced health communication and health promotion efforts. Given that disease manifests from the compound of multiple risk factors—and said factors are differentially distributed across various lifestyles and identities—audience-centered approaches have the potential to be highly effective vehicles for health transformation and useful for any number of audiences, ranging from transgender women to Black men who have sex with men to older persons managing multiple chronic conditions to COVID-19 survivors.

It is a well-established principle of health communication that targeted and tailored communication is more effective than general messages. The approach advocated in this paper—to build audience-centered communication platforms—is a promising approach to develop more cost-effective, engaging, and effective health communication interventions.

## Abbreviations

CoPHII: Collaboration for Population Health Innovation and Improvement
LLILAS: Lozano Long Institute of Latin-American Studies

## References

[1] Tips From Former Smokers. US Centers for Disease Control and Prevention.

[2] Text4Baby.

[3] Evans WD, Wallace JL, Snider J (2012). Pilot evaluation of the text4baby mobile health program. BMC Public Health.

[4] Evans WD, Wallace Bihm J, Szekely D, Nielsen P, Murray E, Abroms L, Snider J (2014). Initial outcomes from a 4-week follow-up study of the Text4baby program in the military women's population: randomized controlled trial. J Med Internet Res.

[5] Making Health Communication Programs Work (Pink Book). National Cancer Institute.

[6] Mackert M, Lazard A, Love B (2017). Designing Effective Health Messages.

[7] Bright by Text.

[8] (2019). Mobile Fact Sheet: Internet/Broadband, Mobile, Social Media. Pew Research Center.

[9] Riley W, Rivera D, Atienza A, Nilsen W, Allison S, Mermelstein R (2011). Health behavior models in the age of mobile interventions: are our theories up to the task?. Transl Behav Med.

[10] Heron KE, Smyth JM (2010). Ecological momentary interventions: incorporating mobile technology into psychosocial and health behaviour treatments. Br J Health Psychol.

[11] Runyan J, Steinke EG (2015). Virtues, ecological momentary assessment/intervention and smartphone technology. Front Psychol.

[12] Nahum-Shani I, Smith SN, Spring BJ, Collins LM, Witkiewitz K, Tewari A, Murphy SA (2018). Just-in-time adaptive interventions (JITAIs) in mobile health: key components and design principles for ongoing health behavior support. Ann Behav Med.

[13] Howell EA (2018). Reducing disparities in severe maternal morbidity and mortality. Clin Obstet Gynecol.

[14] Pérez C, Sánchez H, Ortiz A (2013). Prevalence of overweight and obesity and their cardiometabolic comorbidities in Hispanic adults living in Puerto Rico. J Community Health.

[15] Sanna L, Stuart A, Pasco J, Kotowicz M, Berk M, Girardi P, Brennan S, Williams L (2013). Physical comorbidities in men with mood and anxiety disorders: a population-based study. BMC Med.

[16] Zvara B, Schoppe-Sullivan S, Dush C (2013). Fathers' involvement in child health care: associations with prenatal involvement, parents' beliefs, and maternal gatekeeping. Fam Relat.

[17] Mullany B, Becker S, Hindin M (2007). The impact of including husbands in antenatal health education services on maternal health practices in urban Nepal: results from a randomized controlled trial. Health Educ Res.

[18] Mackert M, Guadagno M, Lazard A, Donovan E, Rochlen A, Garcia A, Damásio M, Crook B (2018). Engaging men in prenatal health via eHealth: findings from a national survey. JMIR Pediatr Parent.

[19] Mackert M, Guadagno M, Lazard A, Donovan E, Rochlen A, Garcia A, Damásio M (2017). Engaging men in prenatal health promotion: a pilot evaluation of targeted e-health content. Am J Mens Health.

[20] Light A, Obedin-Maliver J, Sevelius J, Kerns J (2014). Transgender men who experienced pregnancy after female-to-male gender transitioning. Obstet Gynecol.

[21] Kaye D, Kakaire O, Nakimuli A, Osinde M, Mbalinda S, Kakande N (2014). Male involvement during pregnancy and childbirth: men's perceptions, practices and experiences during the care for women who developed childbirth complications in Mulago Hospital, Uganda. BMC Pregnancy Childbirth.

[22] Deave T, Johnson D (2008). The transition to parenthood: what does it mean for fathers?. J Adv Nurs.

[23] Cabrera N, Shannon J, Mitchell S, West J (2009). Mexican American mothers and fathers' prenatal attitudes and father prenatal involvement: links to mother-infant interaction and father engagement. Sex Roles.

[24] Lee S, Sanchez D, Grogan-Kaylor A, Lee J, Albuja A (2018). Father early engagement behaviors and infant low birth weight. Matern Child Health J.

[25] Cabrera N, Volling B, Barr R (2018). Fathers are parents, too! Widening the lens on parenting for children's development. Child Dev Perspect.

[26] Schmid K, Rivers S, Latimer A, Salovey P (2008). Targeting or tailoring? Maximizing resources to create effective health communications. Mark Health Serv.

